# The Seven Ages of Man

**Published:** 1888-10-20

**Authors:** 


### The Doctor's Pulpit.

### THE SEVEN AGES OF MAN.

### III.—The Lover.

"Then the lover, sighing like furnace."

Nothing better shows the stuff a man is made of than his way of making love. Just as Shakespeare distinctly libelled babies, so he does injustice to the highest type of lover. Possibly he had a sincere contempt for the whole species. The brief description of one of them as "sighing like furnace, with a woeful ballad made to his mistress' eyebrow," is about as contemptuous as it could well be made. Many lovers, it must be confessed, cut a figure which the most sympathetic spectator cannot but regard as ridiculous. The day of "woeful ballads" has gone by; but the spirit which expressed itself in their puerile rhymes remains in full vigour, and will remain so long as man preserves his present physiological characteristics.

But all lovers are not of the "sighing furnace" type, nor are all mistresses pleased with "woeful ballads" made to their eyebrows. Possibly, nay, probably, even in Shakespeare's time there were many lovers whose fineness of feeling raised them above rhyme, or whose strong sense made them regard it with scorn. The lover who finds satisfaction in rhyming is generally a poor creature, and his suit not seldom ends in the "Breach of Promise" Court. All the same, love that has no touch of extravagance is mostly of a poor kind too. Dean Ramsay tells a good story which well illustrates the feminine view of an excessively well-regulated lover. A Scotch pair, after a long period of courtship, are at length brought face to face with the minister, and the knot is securely tied. "Noo, Jean," says the proudly complacent husband, "Hae na I been unco ceevil," alluding to the fact that not once during the whole courtship had he given the fair lady a kiss. "Ou ay," said Jean, "senselessly ceevil." Clearly Jean would have preferred a little more ardour and warmth. We must confess to a sneaking kind of sympathy with the young woman.

Shakespeare, it may be imagined, was not a model lover. In his very early days the present writer devoted much attention to two widely different topics, both however bearing on the marriage question. One was the table of prohibited degrees in the Prayer-book, which used to form a peculiar kind of defence against a long sermon. A boy of ten found a good deal of quiet amusement in the statement that "a man may not marry his grandmother," and so forth. It used to strike him as a piece of more than usually superfluous legislation that a man should be legally prohibited from marrying in that direction. That any man on earth should ever think of marrying his grandame seemed a height of absurdity that no sane person could reach to. Maturer years have dispelled that, as well as some other illusions. The other subject of study was Shakespeare's will. The portion of that very brief document which most excited his wonder and surprise was the sentence in which the dramatist bequeaths to his wife his second best bed. What possible coldness of feeling on a husband's part could make him leave his wife his second best bed? Was it that she was only a kind of second best wife? If she was thus scorned, who was the honoured legatee who became the proud owner of the first best bed? And how many best beds did Shakespeare leave behind? Many a picture of a cold and indifferent husband, somewhat henpecked withal, and of a wife who by no means shared the world's opinion of her lord's greatness, did that will conjure up in the youthful imagination. For, alas! if it be at all true that "no man is a hero to his valet," how much truer is it that no man is a hero to his wife! Whatever be the explanation, it seems certain that the illustrious Stratfordian had a decided contempt for some lovers. For some we say, though not for all. It must not be forgotten that he who poured contempt on woeful ballads written to mistresses' eyebrows created Juliet and Desdemona, Othello and Romeo.

But there is more in love than amusement or food for moralising. It is the profoundest delight of life. But like all other delights, it seems to be surrounded on all sides with limitations, qualifications, risks, and responsibilities, which go far to nullify or completely destroy the happiness it gives. What, for example, is so entirely and indescribably blissful as first love? The very young man sees "heaven in his lover's eyes." The boy and girl who experience the passion for the first time feel an intoxication of rapture such as they never before imagined to be possible. The natural course for them would be to enjoy, without check or hindrance, this beautiful elysium, and in due time to crown and complete their happiness by marriage. But now steps in what the grim parent calls "prudence," "reason," "foresight," and all the thousand and one stony arbiters of destiny which make the world hateful to lovers. The writer cannot but confess a sympathetic wish that circumstances might be made easier for them. It is a hard necessity which compels young people to interminable separation, and to all the agonies of doubt and uncertainty which a long engagement entails. Undoubtedly, the counsellors of prudence and waiting have much right on their sides; but many a young man breathes a most fervent echo to the sentiment expressed by Tennyson so long ago in "Locksley Hall":—

"Cursed be the social wants that sin against the strength of youth;
"Cursed be the social lies that warp us from the living truth."

It is clearly not the intention of Nature that lovers should have so many hindrances put in the way of their happiness: so many impossible mountains to climb before they claim each other for life. An artificial civilisation, which imposes upon men and women an infinity of purely artificial wants is the real barrier which stands in their way. The only gunpowder which will blow up that barrier is of a kind in which youths and maidens are particularly deficient. That gunpowder is self-denial. If young men and women would be content with modest beginnings in the way of house and furniture, of dress, and of friends, they might marry earlier. But hardly any of them will be thus content: and the least contented are generally the feminine half of creation. Women do not seem to be able to do without the dresses, the furniture, and the entertainments which their neighbours enjoy. The only standard of living which will content them is not the standard of reason, or the standard which suits their means; but the standard adopted by their neighbours and friends. Well, but if that be so, there is only one piece of advice to be given, both to them and to young men, and that advice is, "Do not marry." Marriage on insufficient means and an inelastic income is often one of the surest ways to misery and all kinds of bankruptcy which modern civilisation has devised. The father who has seven or eight children and a position to maintain on two or three hundred a-year is not a man to be envied. The mother, having a diminished consciousness of responsibility, may endure her lot better.

Almost unconsciously this paper has taken a rather pessimistic turn. Pessimism, however, ought to be regarded as a baleful enemy by all men, but particularly by lovers. Solomon enjoined upon youthful married couples to be joyful in their married life. Undoubtedly lovers, when they cannot marry immediately, should make the period of their engagement both joyous and hopeful. Although they may wisely look a little way ahead, they should not try to pierce too far into the future. Let them be content with the present. The Englishman's habit of considering how his great grandson is going to get water-cresses with his bread and butter, and Worcester sauce with his cutlets, is just a little foolish. Providence is not dead, and there is a certain very decided advantage in a little homely and reasonable faith.

A closing point in this necessarily discursive paper is well worth making. The point is this, that "constancy" in love is a quality of deep and lasting worth. Fickleness is the mark of a feeble character, and its indulgence tends to blunt the moral sensibilities, as well as to take the bloom off the true happiness of affection. "First love" may not always be prudent, and it may not always be possible to make it "last love." But there is always something peculiarly pleasing and satisfying to the imagination about an aged married couple who began life as boy and girl together, and whose affections have never strayed from each other since the days when they first discovered that they loved one another. Cynicism, which calls itself experience, knowledge of the world, cleverness, and what not, may deride the simplicity which is here held up for approval and practice. But the cynic and the hard man of the world are often but shallow fools. The great facts of nature and of man, of love and domestic peace, of birth and life and death are always with us, and constitute the true landmarks which must always direct our course. Let men and women who would be happy and virtuous love each other, love truly, and, if possible, love but once.

